# Long-term conservation agriculture to enhance soil properties and quality in rice–wheat cropping system

**DOI:** 10.1038/s41598-025-11104-9

**Published:** 2025-08-04

**Authors:** Amit K. Dash, Mahesh C. Meena, Shrila Das, Abir Dey, Seema Sangwan, R. S. Bana, Kaustav Aditya, Md. Basit Raza, Dhaneshwar Padhan, Saloni Tripathy, Tannishtha Bardhan, H. S. Jat, Adarsh Kumar, Nadhir Al-Ansari, Ali Salem, Mohamed A. Mattar

**Affiliations:** 1 ICAR-Indian Agricultural Research Institute (IARI), New Delhi, 110012 India; 2 ICAR-National Institute of Seed Science and Technology, Mau, Uttar Pradesh 275103 India; 3 ICAR-Indian Agricultural Statistics Research Institute, New Delhi, 110012 India; 4 ICAR-Directorate of Floriculture Research, Pune, Maharashtra 411036 India; 5 Central Sericultural Research & Training Institute, Central Silk Board, Mysuru, Karnataka 570008 India; 6 International Corporation Division, Ministry of Agriculture and Farmers Welfare, New Delhi, 110001 India; 7 ICAR-Indian Institute of Maize Research, Ludhiana, Punjab 141008 India; 8 ICAR-Indian Institute of Pulse Research, Kanpur, Uttar Pradesh 208024 India; 9 Department of Civil, Environmental, and Natural Resources Engineering, Lulea University of Technology, 97187 Lulea, Sweden; 10 Civil Engineering Department, Faculty of Engineering, Minia University, Minia, 61111 Egypt; 11 Structural Diagnostics and Analysis Research Group, Faculty of Engineering and Information Technology, University of Pécs, Pécs, 7622 Hungary; 12 Department of Agricultural Engineering, College of Food and Agricultural Sciences, King Saud University, P.O. Box 2460, Riyadh 11451, Saudi Arabia

**Keywords:** Soil quality, Conservation agriculture, Rice–wheat cropping system, Soil organic C stock, System productivity, Agroecology, Agroecology

## Abstract

Conservation agriculture (CA) presents a promising substitute to the tillage-intensive rice–wheat cropping system (RWS) prevalent in the Indo-Gangetic plains (IGPs). In the northwestern IGPs, on-farm studies examining the impact of CA durations on soil properties and quality are limited. This study assessed the effects of CA practised for 2 (CA2), 4 (CA4), 8 (CA8), and 12 (CA12) years and conventional tillage (CT) on soil quality in the Nilokheri block of Haryana, India. The collected soil samples from 0–5 to 5–15 cm were analyzed for 22 different soil parameters, and a soil quality index (SQI) was developed using principal component analysis (PCA) for each scenario. The results showed that scenarios CA8 and CA12 had 9.8–10.7 and 11.1–11.3% lower bulk density, respectively, compared to CT. Mean weight diameter, saturated hydraulic conductivity, and water holding capacity were significantly higher in CA8 and CA12 over CT at both soil layers. Microbial biomass carbon and dehydrogenase activity increased by 32 and 42.7%, 14.9 and 32.3% in CA8 and CA12, respectively, over CT in the surface soil. Most of the chemical parameters were significantly influenced by CA, except for pH, electrical conductivity, and available Cu. Key soil quality indicators identified through PCA included Ks, WHC, β-glucosidase activity, dehydrogenase activity, available S, available Fe, and available Cu. The highest SQI was observed in CA12, followed by CA8 and CA4, and the lowest in CT at both depths. The derived regression coefficients revealed a strong positive relationship between SQI and both rice equivalent yield and wheat yield. This finding highlights the potential of enhancing soil quality to boost agricultural productivity under CA, thereby fostering sustainable farming. Such improvements are vital for building climate-resilient cropping and supporting the widespread adoption of CA practices. Therefore, it may be concluded that adopting CA for more than 8 years could help restore soil health and sustain productivity in the rice–wheat cropping system of northwest IGPs.

## Introduction

The rice–wheat cropping system (RWS), covering approximately 13.5 M ha in the South Asian Indo-Gangetic plains (IGPs) (Bangladesh, India, Nepal, and Pakistan), is one of the most important production systems crucial for ensuring food security and supporting the livelihoods of millions of people^1^. Clean cultivation and frequent tillage in RWS greatly accentuate soil erosion by disrupting soil aggregates and enhancing soil organic carbon (SOC) depletion^2–4^. When coupled with residue burning or removal, these practices further degrade soil health and contribute to environmental pollution^5–8^. Late sowing of wheat in the RWS is a major issue due to the late maturation of rice and improper management of post-harvest residue, thus reducing wheat yield. All these factors lead to yield stagnation and unsustainability of RWS^9–11^. To effectively address these challenges in conventional RWS, conservation agriculture (CA) has emerged as a vital and promising strategy in the IGPs region. As stated by the FAO (Food and Agriculture Organization), CA is a farming system that promotes minimum soil disturbance, continuous soil cover by crop residues, and crop diversification, including the integration of legumes. Numerous previous studies have highlighted the positive impact of CA on soil properties in rice-based systems^12–14^. The CA fosters SOC stabilization and natural biological processes within the soil and enhances soil quality, thereby facilitating sustained crop production^15–17^. Understanding and maintaining soil quality is fundamental to achieving long-term agricultural productivity and environmental health^18–20^. Therefore, to understand the impact of CA on soil quality, it is essential to study the physicochemical and biological characteristics of soil, which are presumed to be changed over extended periods^21^. Integrating these properties into the soil quality index (SQI) provides a comprehensive assessment of soil quality, rather than evaluating specific properties alone. The SQI, calculated using various soil parameters^22^, reflects the dynamic nature of soil properties in response to crop management practices like CA. In the early 1990s, frameworks were developed to estimate SQI using mathematical or statistical methods^23^, linking soil quality to both management practices and inherent soil characteristics. Naorem et al.^24^ stated that eight years of CA practices are likely to show a higher SQI owing to the improvement in several soil quality indicators. Stagnari et al.^25^ reported that seven years of adoption of CA had a notable impact on some soil quality parameters. Significant positive effects of 4 years of CA on biological soil quality parameters were realized in the RWS of central India^26^. The RWS reported improvement in physical soil quality parameters after seven years of adopting zero-tillage in northwestern IGPs^27^. Improvement in the soil quality index was observed after adopting CA for 20 years^28^. However, Bhattacharya et al.^29^ noticed that there were no significant changes in soil properties for three years of CA under RWS in western India.

Therefore, there is a need to understand the time required by conservation agricultural practices to show significant impact on soil quality parameters and, consequently, SQI. Soil quality has been evaluated using the soil management assessment framework (SMAF) following 3-year CA management in a maize-based cropping system on Haplic Cambisol^30^. A biological soil health index, primarily based on soil biological properties, was developed using a scoring method during a ten-year CA experiment in northwestern India^31^. While studies on effect of CA on soil properties have been conducted in other parts of the world, no comprehensive effort has yet been made to evaluate the temporal dynamics of CA adopted for varying durations (short-, medium-, and long-term) on soil quality parameters under the RWS in the unique agro-ecological context of South Asia, particularly in Northwest India. Similarly, very few attempts have been made to monitor changes in SQI due to various durations of CA under the RWS in Northwest India. In other words, on-farm assessment of the effect of short-, medium- and long-term CA on soil quality under the RWS is limited. Furthermore, few studies have combined physical, chemical, and biological properties into a single index to comprehensively assess the overall impact of CA-based RWS on soil quality in the northwestern IGPs. The novelty of this research lies in its on-farm, duration-specific evaluation of soil quality, integrating multiple indicators into a comprehensive SQI using robust multivariate statistical techniques. Unlike previous studies, this work captures the temporal evolution of CA impacts under actual farming conditions, which better reflect the variability and complexity of real-world agricultural systems. So, our concern is “Does CA affect soil quality significantly? If yes, how long does it take to reflect its effect on soil properties and quality? Therefore, we hypothesized: (1) longer durations of CA would greatly enhance the soil physical, chemical and biological properties at 0–5 and 5–15 cm soil depths; (2) Adoption of CA would manifest higher SQI over CT at both soil depths. Therefore, the present study was undertaken on soils collected from farmers’ fields practising CA for 2, 4, 8, and 12 years as well as from conventional tillage (CT) fields with the objective (1) to assess the effect of different durations of CA practices and CT on physical, chemical, and biological properties of soil; (2) to identify key indicators of soil quality for CA and CT in rice–wheat cropping system of northwestern Indo-Gangetic plains; and (3) to compute SQI for CA durations and CT at 0–5 and 5–15 cm soil depths.

## Materials and methods

### Study site and treatments

**Fig. 1:**
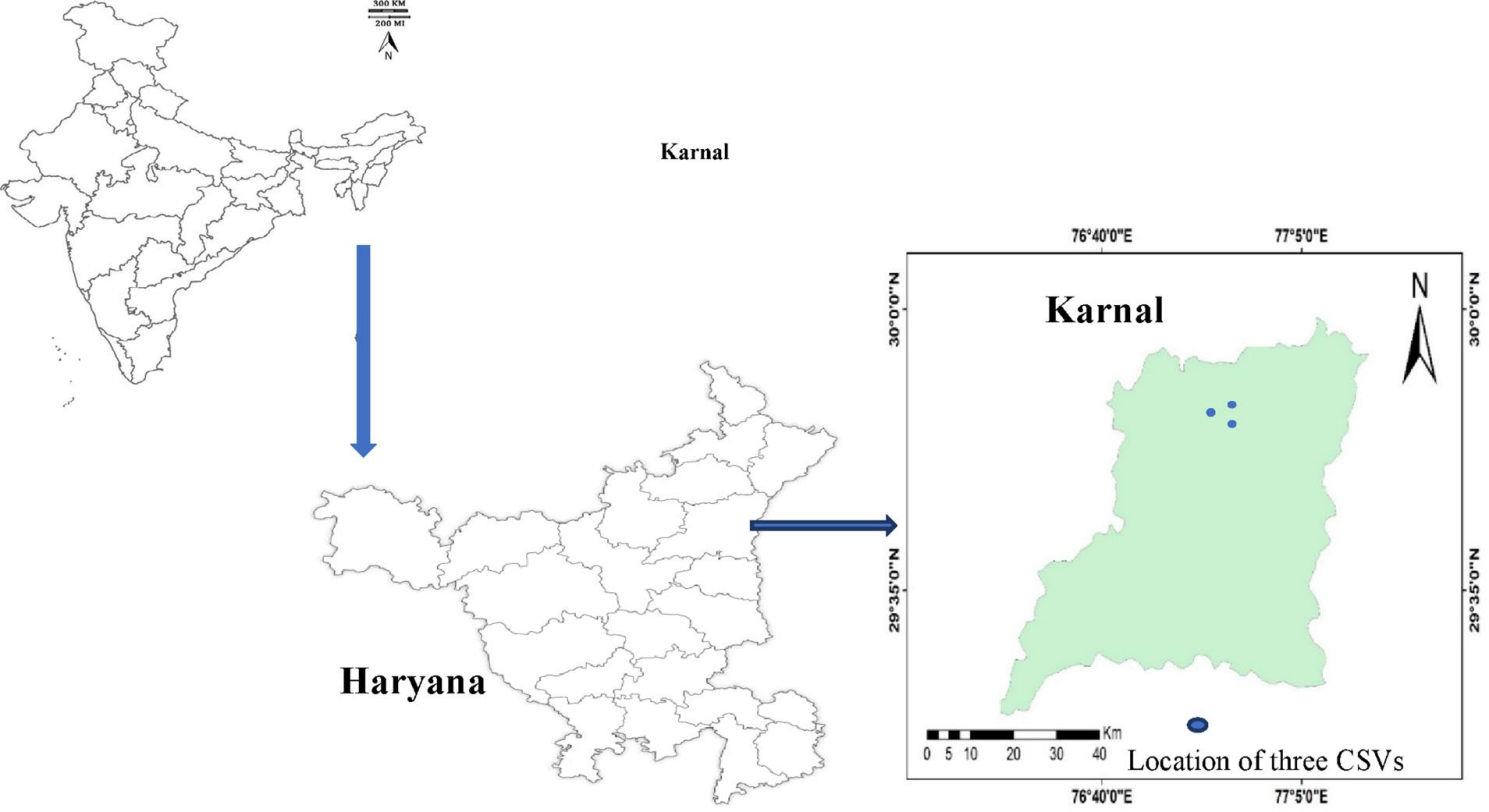
Geographic location of Karnal district (three dots represent three selected villages in Karnal district). (Software used was ArcGIS Desktop version 10.8, https://www.arcgis.com/index.html).

To achieve our research objectives, we initially reviewed existing literature on CA adoption among rice–wheat farmers in the northwestern IGPs, drawing insights from a previous study^32^. Building upon this, a field survey was conducted in 2022 across the Karnal district of Haryana to identify farmers practising CA and assess the duration of their adoption (Fig. 1) (This survey was performed following relevant guidelines and regulations). The survey findings indicated that CA was being practised for varying lengths of time in three villages: Nilokheri, Taraori, and Sambli, situated within the Nilokheri block (Fig. 1). These villages, designated as part of the Climate-Smart Village (CSV) initiative by CIMMYT, India, were deemed ideal locations for soil sampling in this study. The region experiences a subtropical humid monsoon climate, with an annual average rainfall of 506 mm recorded between 2014 and 2022. Average temperature variations range from a low of 9 °C in January to a high of 35 °C in June. Detailed year-wise temperature and rainfall data for the study period (2014–2022) are provided in Table S1. The soil in this area, representative of the northwestern IGPs, exhibits a texture ranging from sandy clay loam to clay loam and is classified under the Entisol order. Farmers practising CA for 2, 4, 8, and 12 years were selected, with these durations considered as distinct scenarios in the study (Table 1). The selected fields, each covering approximately 1000 m^2^, followed a continuous RWS. For each scenario, four replicated soil samples were collected, and data were analyzed using a randomized block design (RBD). The year of adoption for each CA scenario is detailed in Table 1. During the monsoon (kharif) season, direct-seeded rice (cv. Pusa 1121) was cultivated, whereas in the winter (rabi) season, wheat (cv. HD 2937) was sown under zero-tillage conditions using drill seeding (Table S2). As part of CA practices, 30% of the crop residues were retained on the soil surface. At harvest, farmers cut the crops, ensuring that approximately 30% of the plant height remained intact. The recommended fertilization schedule included nitrogen (120 kg ha^−1^), phosphorus (40 kg ha^−1^), and potassium (40 kg ha^−1^). Nitrogen was supplied through urea and diammonium phosphate (DAP), while phosphorus was sourced from DAP and potassium from muriate of potash. The full dose of phosphorus and potassium, along with one-third of the nitrogen, was applied as a basal dose before sowing. The remaining nitrogen was split into two applications: in rice, it was applied at the tillering and panicle initiation stages, while in wheat, it was applied at the crown root initiation and tillering stages.

**Table 1:** Scenario details.

Scenarios	Details	Year of adoption	Category of CA
CA2	2 years of CA practice	2020	Short-term
CA4	4 years of CA practice	2018	Short-term
CA8	8 years of CA practice	2014	Medium-term
CA12	12 years of CA practice	2010	Long-term
CT	Conventional tillage practice	–	–

### Soil sampling and analysis

In the first week of June 2022, quadruplicate soil samples were collected from each scenario at depths of 0–5 and 5–15 cm using a tube auger, following standard soil sampling procedures. The collected soil samples were divided into three sets. The initial batch of fresh, moist soil samples were kept at 4 °C in a fridge for the analysis of biological parameters. The second batch was allocated for examining physical soil properties, including saturated hydraulic conductivity (Ks), water holding capacity (WHC), bulk density (BD), and mean weight diameter (MWD). The third batch was air-dried in the shade, then ground and sieved through a 2 mm mesh to prepare samples to analyze soil chemical parameters. The BD was determined using the core sampling technique described by Blake and Hartge^33^. The wet sieving method was followed to determine MWD^34^. For Ks, the constant head method was used^35^. The WHC was estimated using the Keen-Raczkowskii box method^36^. Among biological parameters, microbial biomass carbon (MBC) was quantified using the chloroform fumigation extraction method as outlined by Vance et al.^37^. For soil enzyme determination, viz., dehydrogenase (DHA) and β-glucosidase (GLUA) activity, colorimetric methods given by Casida et al.^38^ and Eivazi and Tabatabai^39^ were used, respectively. Potentially mineralizable nitrogen (PMN) was assessed by incubating soil samples for 7 days at 40 °C under aerobic conditions, followed by extraction with 4 M KCl^40^. The glomalin content was assessed using the sodium citrate method outlined by Wright and Upadhyaya^41^. The quantification of mycorrhizal spores was performed using the wet sieving and decanting method, detailed by Gerdemann and Nicolson^42^. Soil pH and electrical conductivity (EC) were assessed in a 1:2 soil–water suspension, adhering to the standardized procedure recommended by Jackson^43^. Soil organic carbon (SOC) was determined through wet oxidation using potassium dichromate and sulfuric acid^44^. Soil available macronutrients, viz. N, P, K, and S were determined by protocols given by Subbiah and Asija^45^ Olsen et al.^46^, Jackson^43^, and Chesnin and Yien^47^, respectively. Soil available cationic micronutrients (Fe, Mn, Zn, and Cu) were evaluated post-extraction with DTPA (0.005 M, pH 7.3) solution, using atomic absorption spectrophotometer^48^. The available B content was determined by extracting with hot-CaCl_2_, following the procedure described by Parker and Gardner^49^. The calculation of SOC stock was performed using the provided formula.

SOC stock (Mg ha^−1^) = SOC (g kg^−1^) × Bulk density (Mg m^−3^) × soil depth (m).

### System productivity

The productivity of the rice–wheat system was quantified as rice equivalent yield (REY) using the formula proposed by Das et al.^50^.

$${\text{REY}}\;{\text{of}}\;{\text{ wheat}} = \left( {{\text{wheat yield}} \times {\text{minimum support price of wheat}}} \right)/{\text{minimum support price of rice}}$$

The REY, wheat yield (WY) and rice yield (RY) served as the management goal for evaluating soil quality in this RWS.

### Minimum data set and soil quality index

The soil management assessment framework (SMAF) as described by Andrews et al.^22^ was followed to calculate the SQI using system productivity as a management goal under RWS. A total of 22 soil quality attributes were chosen based on existing research for rice-based cropping systems. Principal component analysis (PCA) was utilized to condense these 22 attributes into a minimum dataset (MDS) of key indicators^22^. Only the PCs with eigen values of 1 or greater, as recommended by Kaiser^51^, were considered for this study. In each PC, factors with absolute weights within 10% of the maximum weight were chosen. The key indicators were selected from each PC based on the highest factor loading and the greater correlation sum among the attributes. Scoring was done using linear scoring function to convert the soil parameters to unitless functions ranging from 0 to 1. The "less is better" approach was followed for BD, whereas the "more is better" approach was followed for the rest of the parameters. Subsequently, the weighted additive, i.e., soil quality index (SQI), was derived by multiplying the weightage of each MDS variable (Wi) from the PCA with its corresponding score (Si). The weight was obtained by dividing the variance (%) of each PC by the cumulative variance (%) for all the selected PCs. The SQI was developed using the following equation.

$${\text{SQI}}\; = \mathop \sum \limits_{{\text{i}} = 0}^{\text{n}} {\text{Wi}} \times {\text{Si}}$$

A higher SQI was found to correlate with improved soil quality and better performance of soil functions. Subsequently, calculations were done to determine the contribution of each key indicator to the SQI. The validation of the SQI was performed by calculating regression coefficients (RCs) against REY, RY, and WY.

### Statistical analysis

Analysis of recorded data was conducted using SPSS version 21.0 for Windows using the RBD function (SPSS Inc., Chicago, USA). The same statistical software was used to perform PCA. Tukey’s honest significant difference (HSD) test was used to confirm significant variations in the attribute means within treatments at a significance level of 5% (*P* < 0.05). PCA biplot and correlation matrix were prepared in R software using FactoMineR and corrplot packages, respectively.

## Result

### Soil physical parameters

Conservation agriculture practices significantly influenced soil physical properties represented by indicators such as BD, MWD, Ks, and WHC (Table 2). The values of BD (up to 15 cm soil depth) did not show any observable change when CT fields were compared to CA practised for 2 years (CA2) and 4 years (CA4). Meanwhile, adopting CA for ≥ 8 years (CA8 and CA12), notably lowered BD compared to CT. The CA12 exhibited 11.1 and 9.8% lower BD at up to 5 cm and 5–15 cm soil depths, respectively, compared to CT, while CA8 showed 11.3 and 10.7% lower BD at the same depths. The order of MWD in various treatments followed the trend, CA12 > CA8 > CA4 > CA2 > CT at both soil depths. CA adopted for 12 years exhibited greater values of MWD in both soil depths, showing an increase of ~ 53–82% over CT plots and ~ 44–48% over CA4 plots. Whereas, when compared with CA practised continuously for 8 years, the values of MWD were ~ 34–48% higher than CA4 and ~ 40.6–59% higher than CT plots. The 0–5 cm, *i.e.,* topsoil, had 0.25–0.28 mm higher MWD than the subsequent depth, *i.e.,* 5–15 cm, irrespective of scenarios. The highest Ks values were observed in CA12, followed by CA8, CA4, CA2, and CT, which showed the lowest value at both soil layers (Table 2). The CA12 and CA8 showed 42.8 and 34.8% more Ks than CT fields at 0–5 cm soil layer. Likewise, at lower 5–15 cm soil depth, CA12 and CA8 showed 81.4 and 61.1%higher Ks than the CT fields, respectively. Greater values of WHC were noted in CA fields at both soil depths showing an increasing trend with the duration of CA practice. Notably, the increase in WHC in CA12 and CA8 treatments was 22.4 and 17.2%, respectively, compared to CT fields at 0–5 cm soil depth; whereas at subsequent soil depth beyond 5 cm, the corresponding increase was 14.7 and 17.2%over CT fields, respectively.

**Table 2:** Effect of conservation agriculture on soil physical parameters in 0–5 and 5–15 cm soil depth.

Scenarios	Soil physical parameters							
	0–5 cm				5–15 cm			
	BD (Mg m^−3^)	MWD (mm)	Ks (cm day^−1^)	WHC (%)	BD (Mg m^−3^)	MWD (mm)	Ks (cm day^−1^)	WHC (%)
CA2	1.41bc	0.68c	54.9c	43.2bc	1.56bc	0.41b	33.3c	39.8bc
CA4	1.37abc	0.73bc	57.5bc	45.8abc	1.52abc	0.48b	40.2bc	43.3abc
CA8	1.29ab	0.90ab	71.2ab	48.6ab	1.41ab	0.62a	50.3ab	45.5ab
CA12	1.27a	0.98a	75.4a	50.7a	1.40a	0.71a	56.3a	47.1a
CT	1.43c	0.64c	52.8c	41.4c	1.58c	0.39b	31.0c	38.8c

Values with similar lower-case letter within a column for each scenario are not significantly different at *P* < 0.05 according to Tukey’s HSD. CA2- Conservation agriculture followed for 2 years; CA4- Conservation agriculture followed for 4 years; CA8- Conservation agriculture followed for 8 years; CA12- Conservation agriculture followed for 12 years; CT- Conventional tillage.

### Soil biological parameters

Conservation agriculture practices significantly impacted soil biological properties such as MBC, DHA, GLUA, PMN, glomalin, and mycorrhizal spore count equally up to 5 cm and subsequent soil depth (Table 3). Significant differences in MBC were only seen in CA8 and CA12 in comparison to conventionally tilled fields. The maximum MBC was recorded in the CA fields cultivated for 12 years, which is 42.7 and 33% higher than the conventionally tilled scenario at 0–5 and 5–15 cm depths, respectively. Conversion of CT lands to CA-based lands significantly enhanced DHA at 0–5 cm and 5–15 cm depths. Twelve years after the conversion of CT fields to CA resulted in 34% higher DHA compared to the former. Adoption of CA ≤ 8 years did not affect DHA in topsoil. However, in the lower depth CA adopted for ≥ 8 years significantly enhanced DHA. At this depth, CA12 had 33.2% more DHA over CT fields. The order of GLUA was CA12 > CA8 > CA4 > CA2 > CT across soil layers and decreased with depth. The CA12 scenario showed 58.7% increase in GLUA over CT. In addition, CA12 had 46.5, 37, and 21% higher GLUA than CA2, CA4, and CA8 in topsoil (0–5 cm). Similarly, CA, adopted for 8 years, reported 27.6% higher GLUA than CT in topsoil. At 5–15 cm soil layer highest and lowest GLUA were observed in CA12 and CT scenarios, respectively.

**Table 3:** Effect of conservation agriculture on soil biological parameters in 0–5 and 5–15 cm soil depth.

Scenarios	Soil biological parameters											
	0–5 cm						5–15 cm					
	MBC (µg g^−1^)	DHA (μg TPFg^−1^ 24 h^−1^)	GLU (μg PNP g^−1^ h^−1^)	PMN (µg g^−1^)	Glomalin (µg kg^−1^)	Spore count (Number 100 g^−1^)	MBC (µg g^−1^)	DHA (μg TPFg^−1^ 24 h^−1^)	GLU (μg PNP g^−1^ h^−1^)	PMN (µg g^−1^)	Glomalin (µg kg^−1^)	Spore count (Number 100 g^−1^)
CA2	211.0c	152.7c	73.0c	10.2 cd	67.3c	1103c	152.7c	141.4bc	38.1bc	5.88 cd	47.1 cd	973c
CA4	224.8bc	164.6bc	78.1bc	11.9bc	74.0bc	1203bc	164.6bc	145.3bc	43.6bc	6.75bc	51.7bc	1055bc
CA8	255.4ab	172.4bc	88.3b	13.4b	82.5ab	1338b	172.4ab	161.7ab	46.9ab	8.02ab	59.6ab	1180ab
CA12	276.2a	199.5a	107.0a	16.8a	104.4a	1545a	199.5a	181.4a	56.7a	9.55a	69.4a	1273a
CT	193.4c	150.0c	69.2c	8.7d	55.4c	1085c	150.0c	136.1c	34.3c	4.72d	39.2d	943c

Values with similar lower-case letter within a column for each scenario are not significantly different at *P* < 0.05 according to Tukey’s HSD. CA2- Conservation agriculture followed for 2 years; CA4- Conservation agriculture followed for 4 years; CA8- Conservation agriculture followed for 8 years; CA12- Conservation agriculture followed for 12 years; CT- Conventional tillage.

The PMN was found higher in CA scenarios in comparison to CT fields and decreased notably with increasing depth. The mean PMN was reported to be 36.6–93 and 43–100% more in CA scenarios than in conventionally tilled scenarios at 0–5 and 5–15 cm, respectively. After practising CA for 4 years, PMN was significantly higher than CT fields. In the case of glomalin, the values for CT, CA2, CA4, CA8 and CA12 were 55.46, 67.36, 74, 82.52, and 104.42 μg kg^−1^ at topsoil depth and 39.21, 47.14, 51.72, 59.61, and 69. 45 μg kg^−1^ at lower soil depth, respectively. The greatest glomalin content was recorded in the CA12 treatment at both soil depths. The content of glomalin in CA8 and CA12 was significantly higher as compared to CT at topsoil and lower depth. CA-based scenarios had significant effect on mycorrhizal spore count. The CA12 and CA8 scenarios were reported to have 42.4 and 23.3% higher spore count over the CT scenario at 0–5 cm. This pattern was also evident at the 5–15 cm soil depth. The mycorrhizal spore count decreased with increasing depth.

### Soil chemical parameters and organic carbon stock

**Fig. 2:**
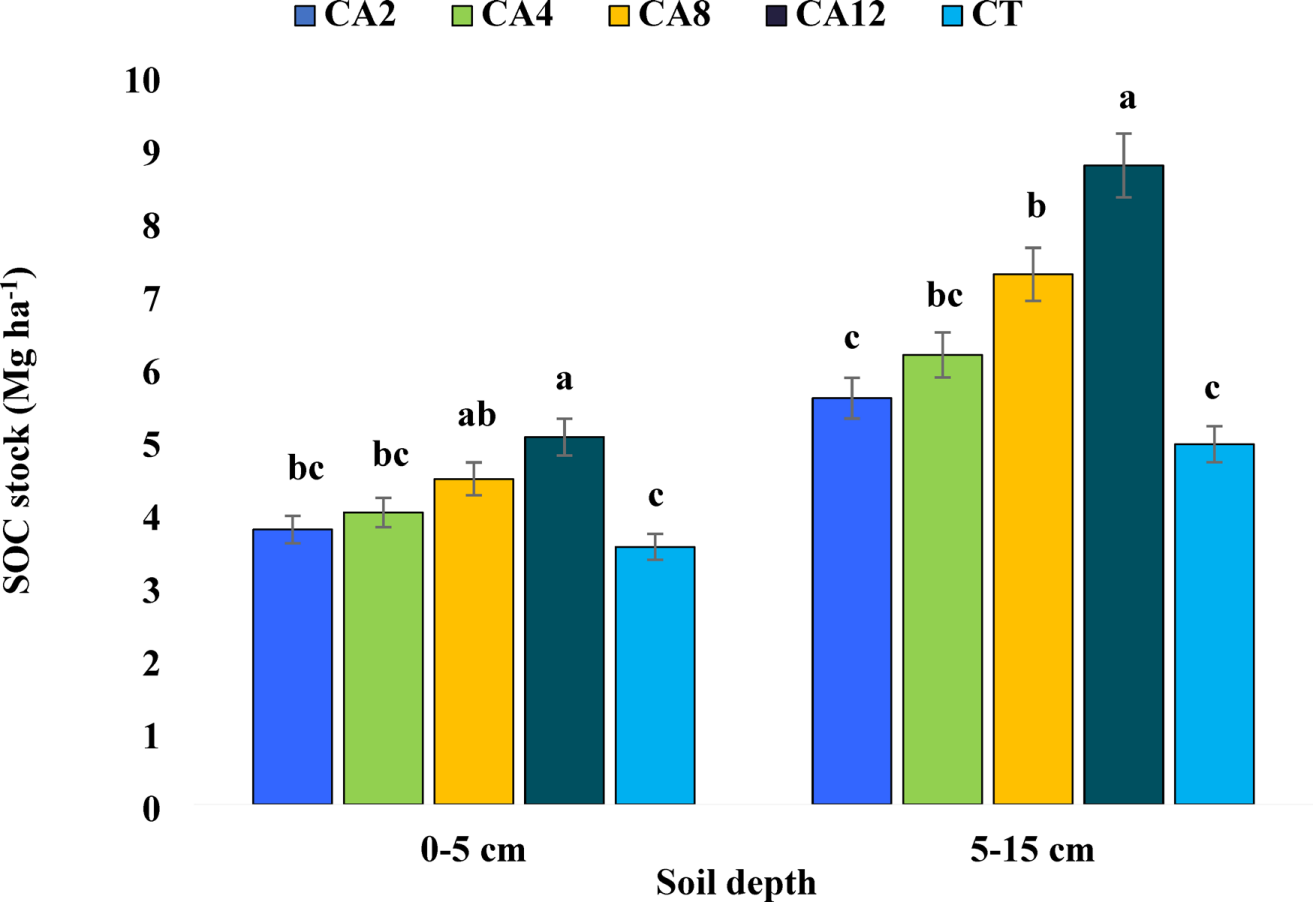
Effect of conservation agriculture on SOC stock in 0–5 and 5–15 cm soil depth. CA2- Conservation agriculture followed for 2 years; CA4- Conservation agriculture followed for 4 years; CA8- Conservation agriculture followed for 8 years; CA12- Conservation agriculture followed for 12 years; CT- Conventional tillage. Values with similar lower-case letter within a column for each scenario are not significantly different at *P* < 0.05 according to Tukey’s HSD between different scenarios.

Conservation agriculture practices significantly influenced soil chemical properties equally at 0–5 cm and subsequent depth 5–15 cm, except pH, EC, and available Cu (Tables 4 and 5). Soil pH ranged from 7.66 to 7.96 in the topsoil (0–5 cm) and from 7.74 to 8.01 at a depth of 5–15 cm across all scenarios. Although practising CA for 2–12 years exhibited slightly lower soil pH compared to CT, the differences at both depths were not statistically significant. Like pH, EC was also not affected by all the CA-based scenarios at all depths. Adopting CA for eight and twelve years significantly boosted SOC at both soil layers, respectively. Transitioning from CT to CA of eight years or more resulted in remarkable SOC increases of ~ 39.47–59.31% in the topsoil and ~ 64.2–98.7% at lower depths. Notably, the topsoil (0–5 cm) consistently contained 1.67–1.83 g kg^−1^ more SOC than the 5–15 cm layer across thescenarios. Similar to SOC content, CA has shown a substantial impact on SOC stock at both soil depths (Fig. 2). The CA12 scenario had 42.37 and 77.5% higher SOC stock over CT at topsoil (0–5 cm) and lower depth (5–15 cm), respectively. However, no differences in SOC content and stock in short-term CA over CT were noticed.

**Table 4:** Effect of conservation agriculture on soil chemical parameters in 0–5 cm soil depth.

Treatments/scenarios	Soil chemical parameters (0–5 cm)											
	SOC (g kg^−1^)	pH	EC (dS m^−1^)	Av. N (mg kg^−1^)	Av. P (mg kg^−1^)	Av. K (mg kg^−1^)	Av. S (mg kg^−1^)	Av. Zn (mg kg^−1^)	Av. Fe (mg kg^−1^)	Av. Mn (mg kg^−1^)	Av. Cu (mg kg^−1^)	Av. B (mg kg^−1^)
CA2	5.35c	7.92a	0.38a	118.4 cd	11.9c	86.9 cd	19.7b	2.28b	22.7bc	17.4bc	1.59a	1.55 cd
CA4	5.84c	7.84a	0.41a	125.0c	13.5bc	95.4bc	21.5ab	2.47ab	23.5abc	18.4abc	1.59a	1.79bc
CA8	6.96b	7.72a	0.34a	142.5b	14.8ab	100.0b	24.3ab	3.22a	27.2ab	21.1ab	1.93a	2.18ab
CA12	7.95a	7.66a	0.38a	169.1a	16.1a	130.5a	26.7a	3.21a	28.1a	22.2a	1.96a	2.53a
CT	4.99c	7.96a	0.40a	108.2d	12.0c	82.7d	18.9b	2.22b	20.1c	16.2c	1.66a	1.27d

Values with similar lower-case letter within a column for each scenario are not significantly different at *P* < 0.05 according to Tukey’s HSD. CA2- Conservation agriculture followed for 2 years; CA4- Conservation agriculture followed for 4 years; CA8- Conservation agriculture followed for 8 years; CA12- Conservation agriculture followed for 12 years; CT- Conventional tillage.

**Table 5:** Effect of conservation agriculture on soil chemical parameters in 5–15 cm soil depth.

Treatments/scenarios	Soil chemical parameters (5–15 cm)											
	SOC (g kg^−1^)	pH	EC (dS m^−1^)	Av. N (mg kg^−1^)	Av. P (mg kg^−1^)	Av. K (mg kg^−1^)	Av. S (mg kg^−1^)	Av. Zn (mg kg^−1^)	Av. Fe (mg kg^−1^)	Av. Mn (mg kg^−1^)	Av. Cu (mg kg^−1^)	Av. B (mg kg^−1^)
CA2	3.57c	7.96a	0.37a	86.6 cd	9.02bc	71.8c	12.0b	1.91ab	19.2bc	14.3 cd	1.45a	1.20bc
CA4	4.05c	7.91a	0.39a	96.9bc	10.7ab	76.8bc	14.5ab	2.02ab	21.0abc	15.6bc	1.49a	1.40abc
CA8	5.19b	7.76a	0.34a	101.9b	11.4a	83.4b	18.6ab	2.16a	22.8ab	17.0ab	1.63a	1.48ab
CA12	6.28a	7.74a	0.36a	120.8a	12.5a	107.7a	22.3a	2.44a	24.4a	18.8a	1.85a	1.71a
CT	3.16c	8.01a	0.39a	83.7d	7.91c	70.0c	12.4b	1.46b	17.4c	13.2d	1.38a	1.05c

Values with similar lower-case letter within a column for each scenario are not significantly different at *P* < 0.05 according to Tukey’s HSD. CA2- Conservation agriculture followed for 2 years; CA4- Conservation agriculture followed for 4 years; CA8- Conservation agriculture followed for 8 years; CA12- Conservation agriculture followed for 12 years; CT- Conventional tillage.

The highest level of available nitrogen (Av. N) was observed in CA12, while the lowest was found in CT across both soil layers. As soil depth increased, Av. N content decreased. Notably, after eight years of CA, Av. N was 31.7% higher in the topsoil and 21.7% higher in the 5–15 cm layer compared to CT. The available phosphorus (Av. P) followed the order CA12 > CA8 > CA4 > CA2 > CT at both soil depths. Embracing CA for 8 and 12 years led to a significant increase in Av. P, with topsoil levels rising by 23.1 and 33.6% over the CT scenario, respectively. Similarly, at the 5–15 cm depth, Av. P surged by 44.8 and 58.9%, respectively. Soil available potassium (Av. K) was significantly higher in CA fields compared to CT fields, with a 17–21% increase in the topsoil for those practising CA for 8 or more years. At the 5–15 cm depth, Av. K increased by 19.1–53.9%. The depth-wise distribution of Av. K followed the pattern: 0–5 cm > 5–15 cm, across the scenarios. Similarly, soil available sulfur (Av. S) was notably greater in CA12 fields compared to CT fields at both depths, with increases of 41% at 0–5 cm and 79.6% at 5–15 cm. As depth increased, Av. S content decreased.

Practising CA for eight or more years significantly enhanced soil micronutrients, except for copper, compared to CT across all soil depths (Tables 4 and 5). Available zinc (Av. Zn) ranged from 2.22 to 3.21 mg kg^−1^ at topsoil (0-5 cm) and 1.46–2.44 mg kg^−1^ at lower layer (5–15 cm). Notably, CA8 exhibited 45% and 48% higher Av. Zn than CT at the top 5 cm and subsequent 10 cm depths (5–15 cm), respectively. Available Cu was not significantly affected by CA scenarios in all soil depths. Available iron (Av. Fe) was significantly higher in fields under CA for eight or more years compared to CT across soil layers. Similarly, soil available manganese (Av. Mn) followed the same trend as Av. Fe, ranging from 16.22 to 22.28 mg kg^−1^ at the top 5 cm depth and 13.26–18.86 mg kg^−1^ at the 5–15 cm depth across all scenarios. Significantly higher soil available B (Av. B) was recorded in CA8 and CA12 scenarios over CT in the topsoil. Highest Av. B was observed in CA12 (2.53 and 1.71 mg kg^−1^) and lowermost in CT (1.27 and 1.05 mg kg^−1^) at topsoil (0–5 cm) and subsequent depth of 5–15 cm, respectively.

### Key indicators and soil quality index for topsoil (0–5 cm)

**Fig. 3:**
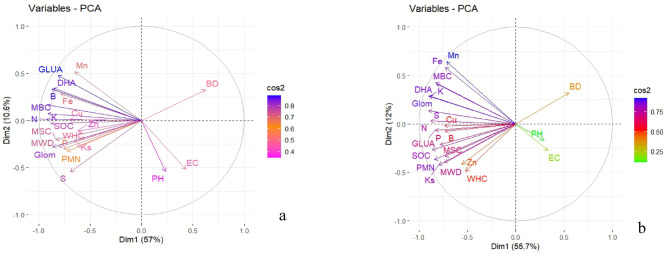
Principal Component Analysis (PCA) biplot based on soil quality attributes as affected by conservation agriculture durations at (**a**) 0–5 cm and (**b**) 5–15 cm depth. BD: Bulk density, Ks: Saturated hydraulic conductivity, WHC: Water holding capacity, MWD: Mean Weight Diameter, pH: Soil pH, EC: Electrical conductivity, SOC: Soil organic carbon, N: Available nitrogen, P: Available phosphorus, K: Available potassium, S: Available sulphur, Zn: Available zinc, Cu: Available copper, Mn: Available manganese, Fe: Available iron, DHA: Dehydrogenase activity, MBC: Microbial biomass carbon, GLUA: Beta glucosidase activity, PMN: Potentially mineralizable nitrogen, Glom: Glomalin content, and MSC: Mycorrhizal spore count.

In this investigation, a comprehensive analysis was conducted on 22 key soil quality parameters using PCA to assess the soil quality indices across various scenarios. Here, from PCA, five PCs having eigen values > 1 were selected, explaining 85% of the total variations in the data set (Fig. 3a and Table 6). In these PCs, GLUA was the highly weighted variable in PC1. Similarly, in PC2, PC3, PC4 and PC5, the highly weighted variables were Ks, WHC, available S and Cu, respectively. These parameters were selected in the MDS because of their higher factor loadings and good correlation with other highly weighted parameters of the respective PCs. After the selection of MDS, we went for linear scoring transformation as given by Sharma et al. (2008). The weighted loadings obtained were 0.320, 0.209, 0.204, 0.158, and 0.110, respectively, for PC1, PC2, PC3, PC4, and PC5. Subsequently, each score was multiplied by its corresponding weight, as determined through the PCA analysis. The SQI for each scenario was computed by aggregating the values of these indicators.

$$\begin{gathered}{\text{SQI}} = \sum {\left[ {\left( {{\text{Glucosidase}}\;{\text{activity}}\;{\text{score}} \times 0.320} \right) + \left( {{\text{Ks score}} \times 0.209} \right)} \right.} \hfill \\\;\;\;\;\;\;\;\;\;\; + \left( {{\text{WHC}}\;{\text{score}} \times 0.204} \right) \hfill \\\left. {\;\;\;\;\;\;\;\;\;\; + \left( {{\text{Av}}.{\text{ S score}} \times 0.158 + {\text{Av}}{\text{. Cu score}} \times 0.110} \right)} \right] \hfill \\ \end{gathered}$$

**Table 6:** Principal component analysis of soil quality attributes as affected by conservation agriculture in the 0–5 and 5–15 cm soil depths.

Parameters	0–5 cm					5–15 cm			
Total	PC1	PC2	PC3	PC4	PC5	PC1	PC2	PC3	PC4
Eigen values	12.532	2.342	1.713	1.156	1.022	12.249	2.633	1.776	1.287
Variance (%)	56.963	10.645	7.788	5.254	4.645	55.676	11.967	8.075	5.848
Cumulative (%)	56.963	67.608	75.396	80.650	85.296	55.676	67.643	75.718	81.566
*Eigen vector*									
SOC	0.460	0.583	0.280	0.384	0.272	0.346	0.677	0.485	0.211
PH	− 0.125	− 0.151	0.053	0.124	− 0.874	− 0.113	− 0.156	− 0.001	− 0.809
EC	− 0.288	0.103	− 0.294	− 0.067	− 0.774	− 0.215	− 0.075	− 0.059	− 0.807
Av. N	0.538	0.457	0.581	0.188	0.200	0.655	0.541	0.279	0.000
Av. P	0.169	0.402	0.652	0.356	0.130	0.443	0.672	0.232	− 0.108
Av. K	0.728	0.349	0.380	0.320	0.006	0.839	0.388	0.199	0.075
Av. S	0.168	0.463	0.234	**0.785**	− 0.208	0.725	0.516	0.213	0.111
Av. Zn	0.245	0.173	0.693	0.125	0.097	0.236	0.693	0.097	− 0.443
Av. Fe	0.829	0.235	0.224	0.158	0.014	**0.935**	0.028	0.204	0.052
Av. Mn	0.912	0.010	− 0.025	0.206	0.096	0.957	− 0.041	0.222	0.079
Av. Cu	0.297	0.139	0.174	0.770	**0.413**	0.371	0.257	**0.737**	0.313
Av. B	0.804	0.314	0.175	0.281	0.251	0.452	0.634	0.039	0.150
BD	− 0.295	− 0.103	− 0.267	− 0.789	0.187	− 0.113	− 0.230	− 0.909	0.121
MWD	0.211	0.597	0.618	0.208	0.146	0.199	0.830	0.176	0.177
WHC	0.231	0.068	**0.874**	0.206	− 0.025	− 0.021	**0.802**	0.024	0.208
Ks	0.273	**0.874**	0.036	0.110	− 0.079	0.207	0.738	0.483	0.135
DHA	0.750	0.320	0.367	0.055	0.280	0.827	0.323	0.055	**0.365**
MBC	0.710	0.343	0.406	0.248	0.161	0.873	0.300	0.075	0.115
Glomalin	0.418	0.525	0.370	0.515	− 0.054	0.823	0.377	0.252	0.141
GLU	**0.913**	0.137	0.226	0.123	0.188	0.522	0.388	0.650	− 0.048
PMN	0.038	0.702	0.538	0.181	0.266	0.344	0.737	0.439	− 0.074
Mycorrhiza	0.400	0.698	0.335	0.249	0.037	0.214	0.684	0.359	0.201

Bold value represents highly weighted attribute within each PC. BD: Bulk density, Ks: Saturated hydraulic conductivity, WHC: Water holding capacity, MWD: Mean Weight Diameter, pH: Soil pH, EC: Electrical conductivity, SOC: Soil organic carbon, N: Available nitrogen, P: Available phosphorus, K: Available potassium, S: Available sulphur, Zn: Available zinc, Cu: Available copper, Mn: Available manganese, Fe: Available iron, DHA: Dehydrogenase activity, MBC: Microbial biomass carbon, GLUA: Beta glucosidase activity, PMN: Potentially mineralizable nitrogen, Glom: Glomalin content, and Mycorrhiza: Mycorrhizal spore count.

**Fig. 4:**
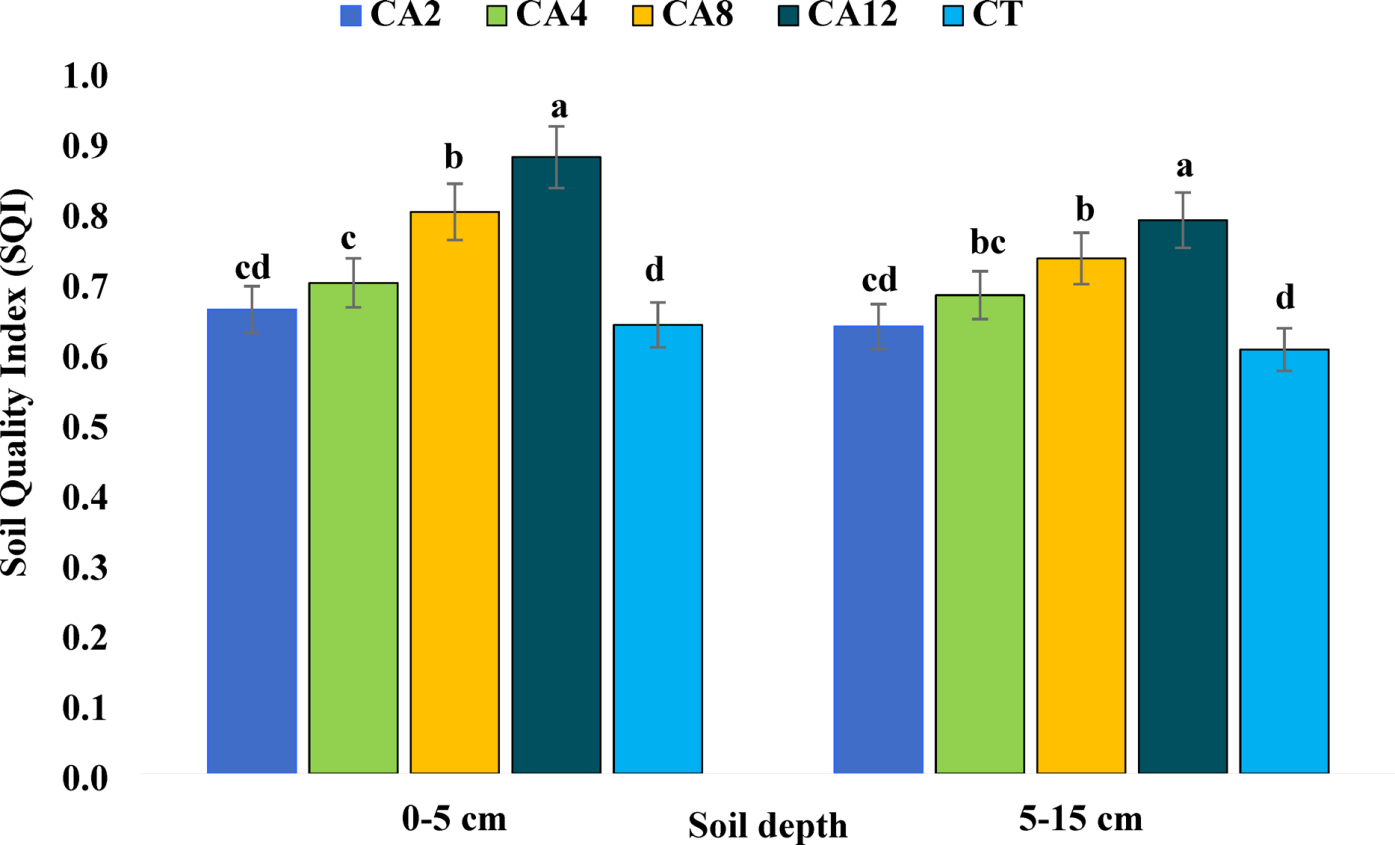
Effect of conservation agriculture on soil quality indices in 0–5 and 5–15 cm soil depth. CA2- Conservation agriculture followed for 2 years; CA4- Conservation agriculture followed for 4 years; CA8- Conservation agriculture followed for 8 years; CA12- Conservation agriculture followed for 12 years; CT- Conventional tillage. Values with similar lower-case letter within a column for each scenario are not significantly different at *P* < 0.05 according to Tukey’s HSD between different scenarios.

**Fig. 5:**
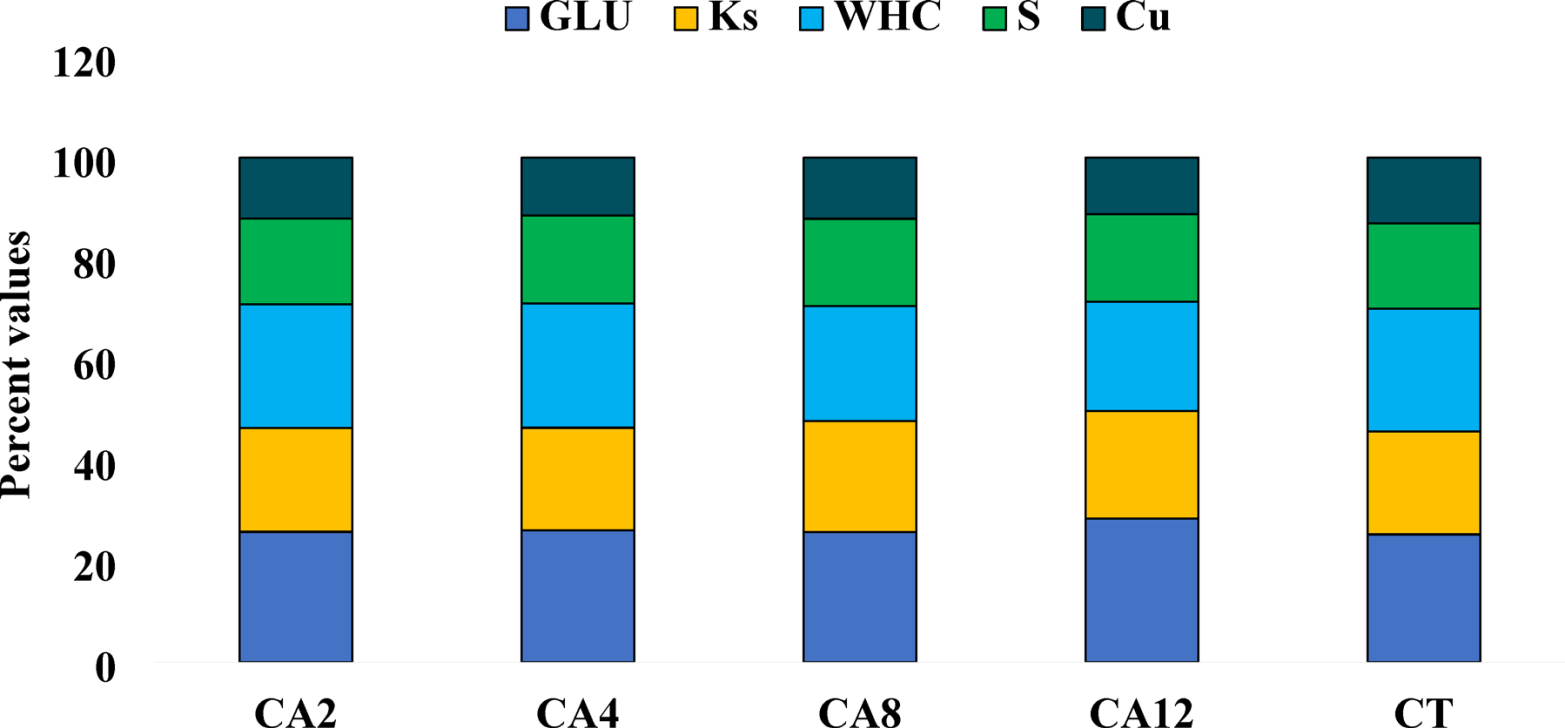
Per cent contributions of key indicators to soil quality indices in the 0–5 cm soil depth. CA2- Conservation agriculture followed for 2 years; CA4- Conservation agriculture followed for 4 years; CA8- Conservation agriculture followed for 8 years; CA12- Conservation agriculture followed for 12 years; CT- Conventional tillage.

The SQI was significantly influenced by the conservation agricultural practices at 0–5 cm soil depth (Fig. 4). The highest SQI was observed in the CA12 scenario, and the lowest in CT. Thus, the trend of SQI observed was CA12 > CA8 > CA4 > CA2 > CT. The SQI of all CA scenarios were significantly better than CT except for the CA2 scenario. The contribution percentage of each indicator to the SQI was also determined (Fig. 5). The highest contribution was by GLU (25.84–28.53%), followed by WHC (21.64–24.56%), Ks (20.37–22.03%), Av. S (16.87–17.7%), and Av. Cu (11.23–13.04%) across the scenarios in the topsoil.

### Key indicators and soil quality index for 5–15 cm soil depth

In the lower soil depth (5–15 cm), PCA was performed on above mentioned 22 key soil quality parameters. But here, only four PCs having eigen values > 1 were extracted, which explained 81.5% of the total variance in the data (Fig. 3b and Table 6). The maximum weighted variables were Av. Fe, WHC, Av, Cu, and DHA in PC1, PC2, PC3, and PC4, respectively and selected as sensitive indicators in MDS. Then they underwent linear scoring. The weighted values calculated were 0.383, 0.336, 0.186, and 0.113, respectively, for PC1, PC2, PC3, and PC4. The SQI was calculated by multiplying each score by its corresponding weight and then summing the value of the results for each indicator.

$$\begin{aligned}{\text{SQI}}& = \sum {\left[ {\left( {{\text{Av}}.{\text{Fe}}\;{\text{score}} \times 0.383} \right)} \right.} + \left( {{\text{WHC}}\;{\text{score}} \times 0.336} \right) \hfill \\& \left. \quad{ + \left( {{\text{Av}}.{\text{Cu}}\;{\text{score}} \times 0.186} \right) + \left( {{\text{DHA}}\;{\text{score}} \times 0.113} \right)} \right] \hfill \\ \end{aligned}$$

**Fig. 6:**
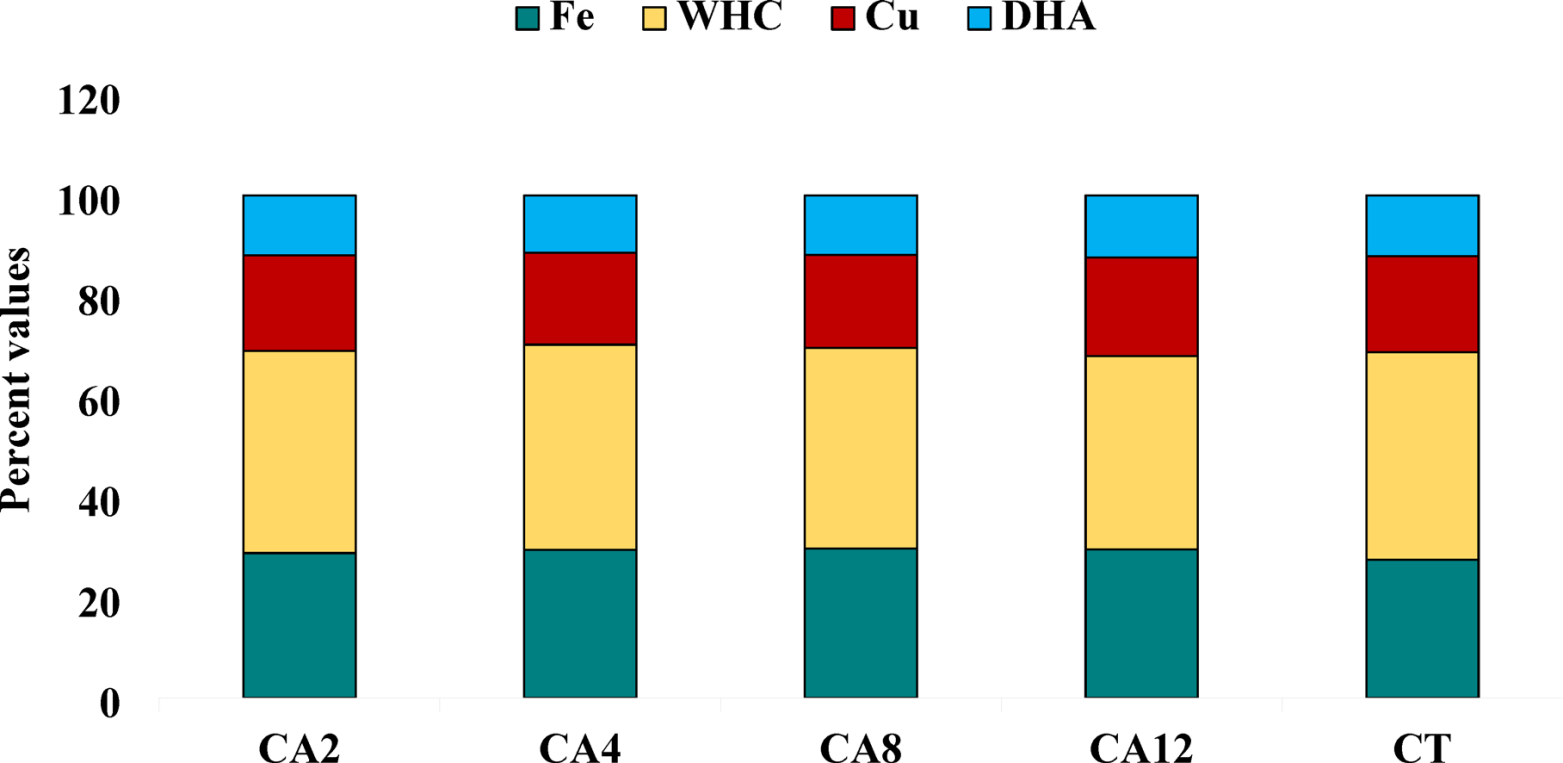
Per cent contributions of key indicators to soil quality indices in the 5–15 cm soil depth. CA2- Conservation agriculture followed for 2 years; CA4- Conservation agriculture followed for 4 years; CA8- Conservation agriculture followed for 8 years; CA12- Conservation agriculture followed for 12 years; CT- Conventional tillage.

At the lower depth (5–15 cm), different CA scenarios had a noteworthy impact on the SQI (Fig. 4). The highest SQI was recorded in CA12, followed by CA8, CA4, CA2, and CT. Like topsoil, SQI values were considerably higher in all CA scenarios compared to CT, except for CA2. The highest contribution was by WHC (38.28–41.26%), followed by Av. Fe (27.5–29.76%), Av. Cu (18.26–19.66%) and DHA (11.46–12.37%) in SQI across the scenarios (Fig. 6).

### Validation of SQI

**Fig. 7:**
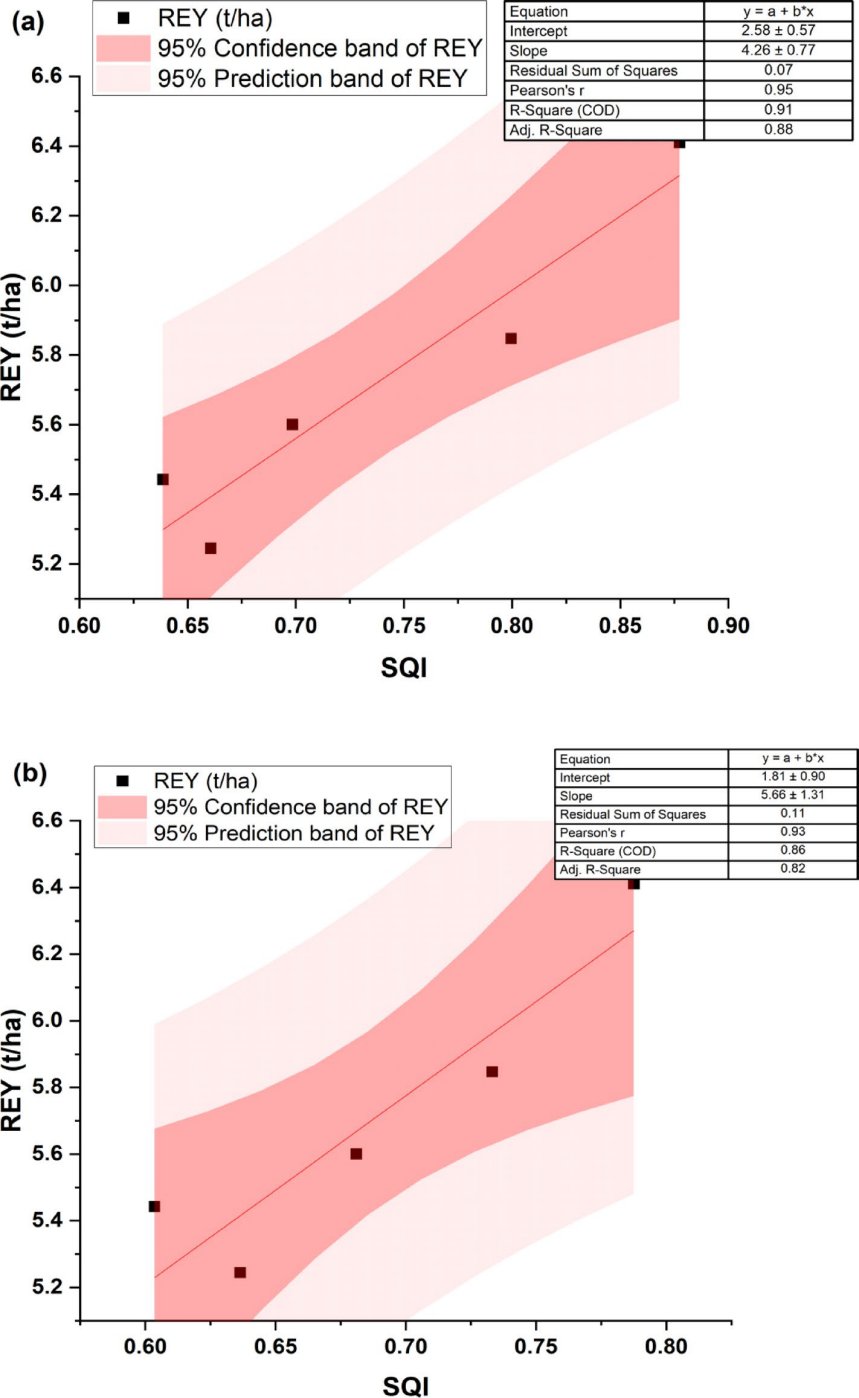
Relationship between SQI and REY under the rice–wheat system at (**a**) 0–5 cm and (**b**) 5–15 cm. SQI- Soil quality index and REY- Rice equivalent yield.

**Fig. 8:**
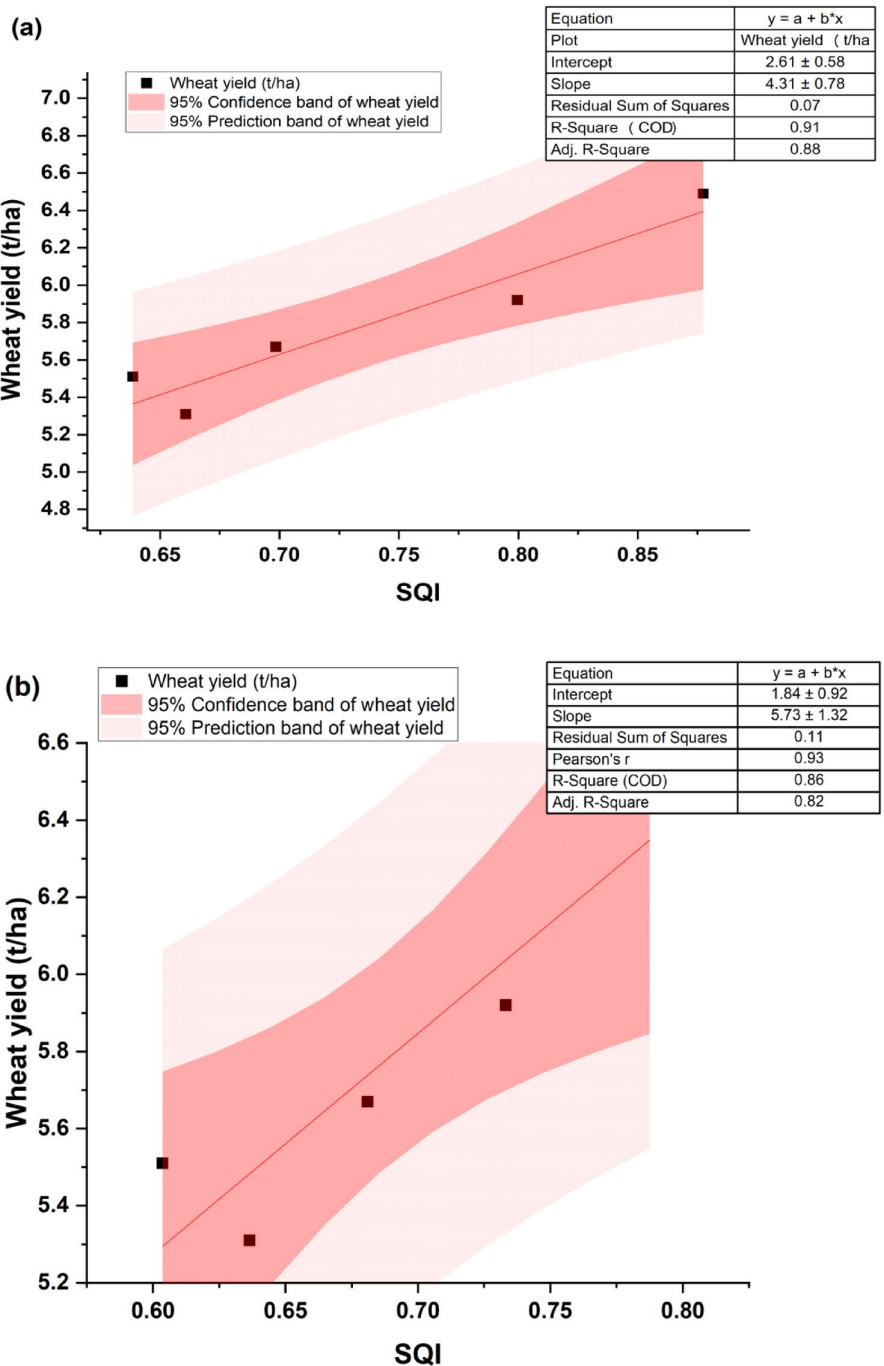
Relationship between SQI and WY under the rice–wheat system at (**a**) 0–5 cm and (**b**) 5–15 cm. SQI- Soil quality index.

**Fig. 9:**
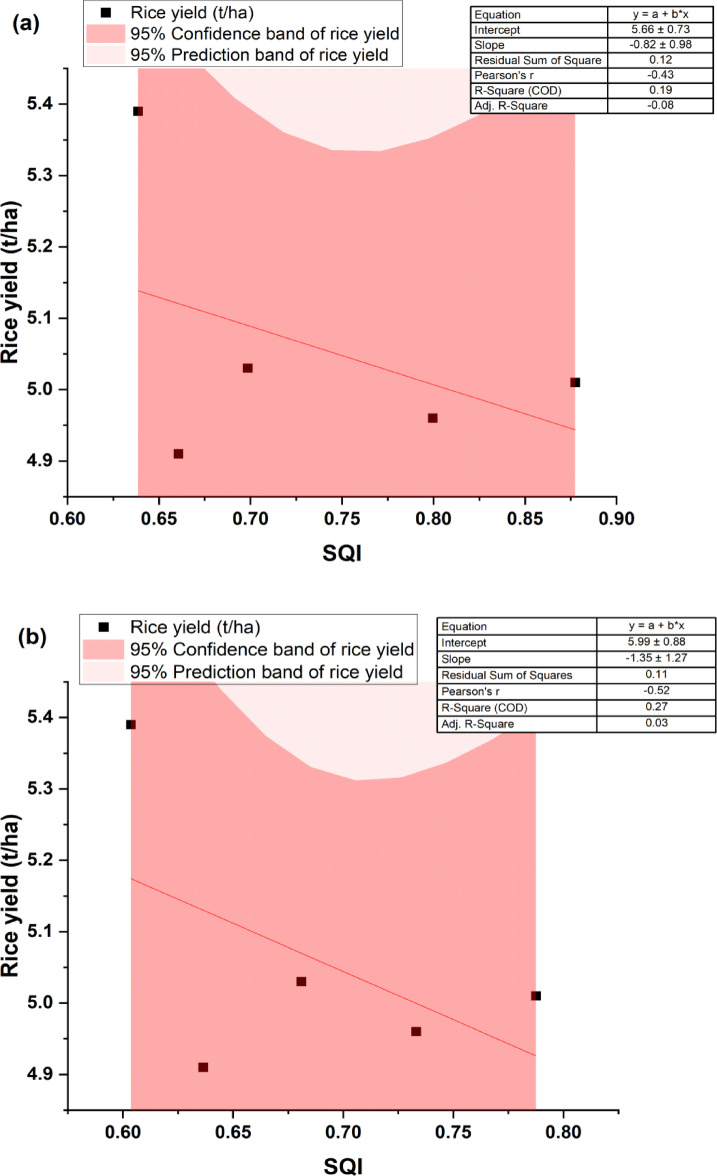
Relationship between SQI and RY under the rice–wheat system at (**a**) 0–5 cm and (**b**) 5–15 cm. SQI- Soil quality index.

The resulting SQI was validated against REY, RY, and WY at both soil depths. In our study, crop yields of 2022–23 were considered. To develop the regression equation, REY, WY, and RY were taken as dependent variables, while SQI was the independent variable under different scenarios for each soil depth. The derived RCs were significant, with R^2^ values of 0.91 and 0.93 at the 0–5 and subsequent soil depth (5–15 cm) for REY, respectively (Fig. 7a and b). Similarly, for WY, significant RCs were estimated with R^2^ values of 0.91 and 0.86 for the 0–5 and 5–15 cm soil layers, respectively (Fig. 8a and b). However, the RCs were not significant for RY, with R^2^ values of 0.19 and 0.27 for the 0–5 cm and subsequent soil depth (5–15 cm), respectively (Fig. 9a and b).

### Correlation between SOC and soil properties

**Fig. 10:**
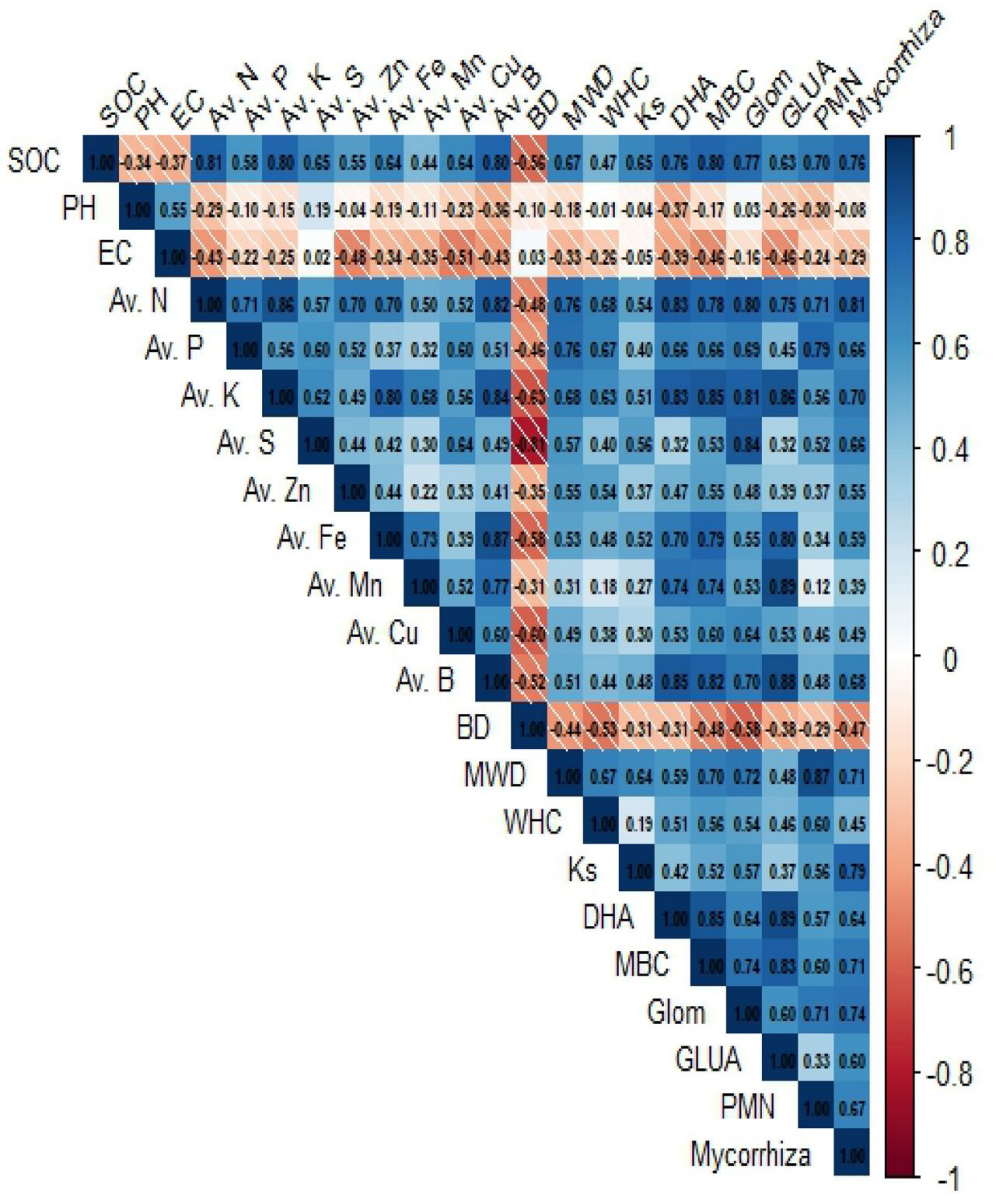
Correlation between all soil quality attributes at 0–5 cm soil depth under CA practices. BD: Bulk density, Ks: Saturated hydraulic conductivity, WHC: Water holding capacity, MWD: Mean Weight Diameter, pH: Soil pH, EC: Electrical conductivity, SOC: Soil organic carbon, N: Available nitrogen, P: Available phosphorus, K: Available potassium, S: Available sulphur, Zn: Available zinc, Cu: Available copper, Mn: Available manganese, Fe: Available iron, B: Available boron, DHA: Dehydrogenase activity, MBC: Microbial biomass carbon, GLUA: Beta glucosidase activity, PMN: Potentially mineralizable nitrogen, Glom: Glomalin content, and Mycorrhiza: Mycorrhizal spore count.

**Fig. 11:**
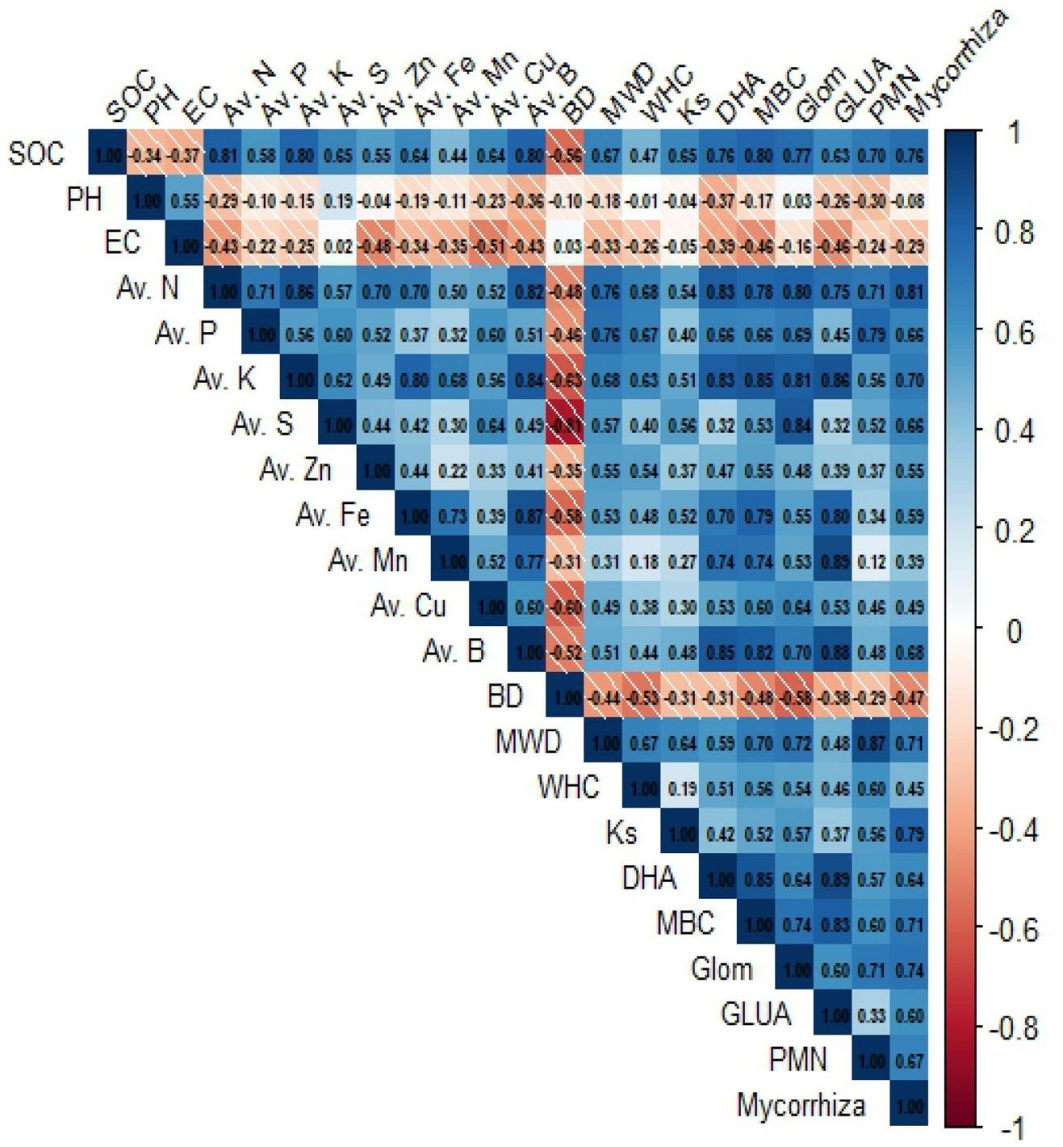
Correlation between all soil quality attributes at 5–15 cm soil depth under CA practices. BD: Bulk density, Ks: Saturated hydraulic conductivity, WHC: Water holding capacity, MWD: Mean Weight Diameter, pH: Soil pH, EC: Electrical conductivity, SOC: Soil organic carbon, N: Available nitrogen, P: Available phosphorus, K: Available potassium, S: Available sulphur, Zn: Available zinc, Cu: Available copper, Mn: Available manganese, Fe: Available iron, B: Available boron, DHA: Dehydrogenase activity, MBC: Microbial biomass carbon, GLUA: Beta glucosidase activity, PMN: Potentially mineralizable nitrogen, Glom: Glomalin content, and Mycorrhiza: Mycorrhizal spore count.

Pearson’s correlation between all the soil quality parameters was studied at 0–5 and 5–15 cm soil depths (Figs. 10 and 11). The SOC had a significant positive correlation (*P* ≤ 0.01 and *P* ≤ 0.05) with all physical, chemical and biological properties except pH, EC, and BD at both soil depths. Among physical parameters, BD was negatively correlated with MWD, Ks, and WHC at both soil depths (Figs. 10 and 11). However, in chemical parameters, pH and EC were negatively correlated to all other soil parameters, showing their adverse impact on overall soil quality.

## Discussion

### Effect of durations of conservation agriculture on soil physical properties

The adoption of CA continuously for eight long years resulted in the substantial addition of crop residues (rice and wheat in our study) to the soil. During the decomposition process, polysaccharides and organic acids are released, which aid in binding soil particles into aggregates^5,52,53^. This process contributes to increased soil porosity and reduced BD at both soil depths. The significant reduction in soil BD observed after eight years of CA in our study aligns with the findings of Osunbitan et al.^54^, who reported similar results compared to CT. Additionally, BD was negatively correlated with SOC (Figs. 3, 10 and 11). Similarly, Enfors et al.^55^ noted that CA adoption for 4–5 years did not affect BD in the topsoil, aligning with the results of our study. In MWD significant effect of CA was visible after 8 years of adoption, which corroborates the findings of Das et al.^50^, where a 28% increase in MWD was reported after 8 years of CA practice. Such result is likely due to reduced soil disturbance and the addition of organic residues associated with CA, which aids in forming stable soil aggregates, shielding them from raindrop impact, and promoting fungal root networks, resulting in higher MWD than CT. This was further confirmed by the positive correlation between SOC and MWD (Figs. 3, 10 and 11). In contrast, CT often causes soil aggregate loss through frequent mechanical disruption and organic matter oxidation^56^. The increased Ks observed in CA could be attributed to enhanced macropore formation and improved pore continuity due to greater SOC accumulation and reduced soil disturbance compared to CT. This aligns with the results reported by Parihar et al.^27^ and Das et al.^50^. After four and six years of transitioning to CA, the WHC of soil remained unchanged as reported by Vogeler et al.^57^ and Alam et al.^58^. However, a study in Spain found a significant increase of 30 and 9.6% in WHC after eight years of CA adoption^59^, which is in correspondence with our findings. Our study confirms that the improvement in WHC soil under 8–12 years of CA practice is brought by the increased SOM content which caused alterations in pore connectivity and pore size distribution, allowing greater water movement and storage.

### Effect of durations of conservation agriculture on soil chemical properties

Short-term CA practices, spanning less than four years, exhibited no significant changes in SOC relative to CT. However, the prolonged adoption of CA, exceeding eight years, notably augmented both SOC content and stock across soil depths as compared to CT plots. Roy et al.^60^ corroborated these findings, reporting elevated SOC and stock levels by 41–57 and 70–90%, respectively, compared to CT after a decade of CA adoption. Noteworthy enhancements in SOC storage were observed in a RWS in South Asia under CA practised for 9 years^61^. Traditional tillage exposes SOC to aeration, escalating organic carbon oxidation. Conversely, reduced tillage under CA fosters organic carbon accumulation^62^. Moreover, crop residue retention significantly augments SOC levels, exemplified by Dash et al.^5^ who recorded nearly two-fold SOC augmentation in plots with 4 t ha^−1^ of crop residue versus those without crop residue, underscoring the pivotal role of its management in enhancing SOC for sustainable agriculture. The reduction in soil pH observed in CA compared to CT could potentially be attributed to the generation of organic acids during the decomposition of crop residues deposited on the soil surface. This process results in the release of protons, thereby slightly lowering pH levels^63^. The result of our study regarding EC is in agreement with the findings of Roy et al.^60^, where EC was similar across the scenarios.

The impact of CA on soil N content closely mimics that observed for SOC, given the interconnectedness of nitrogen and carbon cycles, both intricately linked to SOM^64^. Continued adoption of CA, exceeding four years, has been found to boost SOM accumulation, largely attributed to the steady deposition of crop residues, thus leading to greater concentrations of available N in rice-based cropping systems^65^. This observation aligns with Dey et al.^66^, where a 9% increase in total N was documented under CA-based RWS compared to CT. Moreover, the enhanced protection of micro-aggregate-associated N within macro-aggregates under CA may contribute to increased N availability^67^. Nonetheless, it is important to acknowledge that certain studies suggest CA could potentially decrease soil N levels due to the high carbon-to-nitrogen (C:N) ratio of cereal straw, potentially leading to mineral N immobilization^64^. In topsoil, four years of CA had no significant impact on soil available P. This could be due to insufficient residue accumulation within this timeframe. Conversely, CA practices exceeding eight years led to significant changes in P which could be attributed to organic acids released during the residue decomposition causing solubilization of indigenous P. Fresh residue retention and limited soil mixing of applied P reduced the fixation, adsorption, and precipitation of soluble P, thereby encouraging the formation of soluble phosphate-humate complexes and enhancing P availability. Conversely, extensive soil mixing under CT reduces P availability^68^. The rise in available K concentration noted under CA across soil depths may result from the incorporation of K through crop residues^69^. Das et al.^50^ demonstrated that CA plots with rice residues showed increased levels of available K, attributed to an annual addition of 175 kg K ha^−1^ through rice residues.

After 12 years of CA adoption, significantly higher available S levels in soils were observed compared to CT, likely due to residue incorporation, resembling the findings of Kumar et al.^70^. They observed positive correlations between S fractions and SOC, while also identifying negative correlations with BD, which align with our findings (Figs. 3 and 10). Reduced soil disturbance and increased crop residue retention likely contributed to increased S pools in soils, primarily through enhanced SOC sequestration. Practising CA for over eight years significantly impacted soil micronutrients except for Cu. Our findings follow Franzluebbers and Hons^71^, who observed a notable increase in soil micronutrient levels following nine years of CA. The faster mineralization of micronutrients from SOM under CT likely results in quicker uptake by plants, leading to lower availability afterwards in CT compared to reduced tillage practices^72^. Moreover, Govaerts et al.^73^ found no significant difference in Cu availability between CA and CT in the 0–5 cm soil layer. In longer-duration CA, the increased deposition of organic matter significantly enhances B availability, as organic matter is the primary soil constituent influencing its accessibility. This relationship is substantiated by robust correlations between available B and soil organic matter^74^.

### Effect of durations of conservation agriculture on soil biological properties

Greater MBC in CA after 8 years of adoption was mainly due to higher SOC content over CT. The study by Roy et al.^60^ corroborated our results, reporting 177–192 and 74–102% higher MBC over CT at 0–5 and 5–15 cm soil depth after practising CA for 10 years. No soil disturbance and crop residue retention at surface soil in CA facilitates gradual residue decomposition, releasing labile organic matter and sustaining microbial biomass^75^. This is also justified by the significant positive correlation between SOC and MBC (Table 6). Baghel et al.^76^ observed higher MBC in CA under the RWS, attributing it to moderated soil temperature, prolonged moisture retention, and stable carbon sources for microorganisms. The positive effect of CA practice on DHA was noticed after 12 years. Bhattacharya et al.^29^ reported that adopting CA for more than 9 years resulted in 66. 6% more DHA over CT at 0–15 cm soil depth in RWS. Pratibha et al.^77^ also recorded significantly higher DHA in CA, followed for 11 years. This may result from increased MBC and SOC levels. Our study also found a positive correlation between DHA and SOC (Figs. 10 and 11). The absence of soil disturbance along with residue retention may enhance soil moisture availability to microorganisms, possibly contributing to high DHA under CA^78^. At soil depths of 0–5 and 5–15 cm, the increased GLUA under long-term CA was attributed to the greater organic matter content facilitated by residue retention and the absence of soil disturbance. Our findings accord with those reported by Stott et al.^79^, Roy et al.^60^, and Naorem et al.^24^.

The long-term adoption of CA demonstrated significant effect on PMN levels. A meta-analysis by Mahal et al.^69^ supported our result, where they reported 13% more PMN under long-term CA in surface soils compared to CT. This enhancement is intricately linked to SOM, where the covalent bonds between organic carbon and nitrogen play a crucial role in regulating their mineralization processes, which is a common phenomenon in CA fields^80^. A strong positive correlation between PMN and SOC demonstrated the role of SOC in nitrogen mineralization (Figs. 10 and 11). The soil in CA fields of 8 and 12 years exhibited significantly higher glomalin content and mycorrhizal spore counts compared to CT. Such a result aligns with that of Singh et al.^81^, where elevated glomalin and mycorrhizal spore counts were observed in a direct seeded rice-zero tilled wheat system relative to CT. This might be attributed to higher SOC content in CA, as our study also observed a strong positive correlation between SOC and both glomalin and spore count. CA systems foster a greater population of arbuscular mycorrhizal fungi (AMF), while CT systems harm AMF populations, possibly due to hyphal network disturbance during ploughing operations. This disparity in AMF density could explain the lower glomalin content in CT soils, exacerbated by high macroaggregate turnover rates leading to glomalin decomposition and loss.

### Key indicators and soil quality indices

The sensitive key indicators for soil quality indices were Ks and WHC among physical parameters, GLUA and DHA among biological parameters, and available S, Fe and Cu among chemical parameters for both soil depths. Saturated hydraulic conductivity as a dynamic property holds significant sway in governing the balance between soil moisture and air, particularly in the arrangement of soil pores and ensuring the continuity of macropores^82^. Soil WHC is another physical indicator key to crop production^83^. Enhanced soil WHC is primarily linked to higher infiltration rates and reduced runoff, thus reducing soil erosion severity^84^. Nevertheless, soils characterized by a low WHC experience substantial loss of irrigation or rainfall through deep percolation. Consequently, this leaches nutrients away from the root zone, resulting in inefficient resource utilization, environmental issues, and diminished economic returns^83^. This ascertains the importance of periodic monitoring of soil parameters such as Ks and WHC.

The dehydrogenase enzyme is a key biological indicator, crucial in facilitating the biological oxidation of SOM by transferring protons and electrons from substrates to acceptors. They represent a key component of soil enzymatic activities, ensuring the right sequence of biochemical pathways within soil biogeochemical cycles^85^. So, measuring changes in DHA provided a useful indicator of soil quality. β-glucosidase is responsible for breaking β-1,4 bonds to generate glucose from β-glucosides, a vital process in terrestrial carbon cycling that involves the decomposition of crop residues^79^. Thus, this enzyme plays a direct role in maintaining soil health. One of the chosen chemical indicators was available S, a vital component of proteinaceous amino acids like methionine and cysteine, as well as glutathione, vitamins (biotin and thiamine), phytochelatins, and chlorophyll^86^. Sulphur enhances rice and wheat yields and improves grain quality. It also facilitates the uptake of N, P, and K by rice and wheat plants. Conversely, sulfur deficiency can result in reduced plant height, fewer tillers, decreased panicle formation, and lower grain production^87^. Another important indicator is available Fe, crucial for plant growth due to its involvement in several physiological and biochemical processes. It is a key component of various essential enzymes, including cytochromes in the electron transport chain, and plays a role in chlorophyll synthesis^88^. Copper is a vital metal for plants, involved in photosynthetic and respiratory electron transport chains, ethylene sensing, cell wall metabolism, protection against oxidative stress, and the formation of the molybdenum cofactor. Consequently, a Cu deficiency can disrupt essential metabolic functions in plants^89^.

The CA practices followed for more than four years significantly affected SQI at both soil depths. The SQI was highest at the surface soil (0–5 cm) in all scenarios, likely due to the increased SOC in the surface layer compared to subsurface layers, which positively impacts other soil quality indicators^50^. Our result conformed with Adak et al.^17^, where significantly higher SQI was reported in a four year old CA over CT. Similar improvements in soil quality under conservation tillage were reported in a few recent studies by Stagnari et al.^25^, Roy et al.^60^, and Naorem et al.^24^. The comparatively higher soil quality observed under CA suggests improvement in overall soil properties in the long run.

### Validation of SQI

The significant relationship between SQI and system equivalent yield at both soil depths was reported earlier by many investigators^27,50,90^ in cereal-based systems. In the northwestern IGPs regions, reduction in DSR yield has been reported^50^. Reduced yields in the northwestern IGPs regions may stem from increased weed and nematode infestations, intermittent moisture stress in aerobic rice cultivation, limited understanding of water and nutrient management, and heightened spikelet sterility^50^. Yet, the notable correlations between SQI and both REY and WY provide valuable means for predicting crop yields in response to SQI fluctuations.

### Implications of the study

This study underscores the importance of long-term CA for improving soil health and sustaining productivity in the RWS of the northwestern IGPs. Significant improvements in soil physical, biological, and chemical properties were observed only after eight years of CA adoption, highlighting the need for long-term commitment to these practices. Developing and validating an SQI strongly correlated with crop productivity, suggesting its utility as a practical tool for monitoring soil health and predicting yield. Key indicators such as Ks, WHC, and enzyme activities can serve as early-warning metrics for soil degradation. These findings have direct implications for farmers, researchers, and policymakers, emphasizing the need to support and scale long-term CA practices to ensure sustainable and climate-resilient agriculture in the region.

## Conclusion and recommendation

The results of our study showed significant improvement in soil physical, biological, and chemical properties after eight years of CA adoption at 0–5 and 5–15 cm depths, in the farmers’ fields of northwestern Indo-Gangetic plains (IGPs), confirming the first hypothesis. To have a visible impact on soil BD, CA should be adopted for a period exceeding eight years. However, soil pH, EC, and available Cu didn’t show significant changes under CA even after 12 years of adoption. The adoption of CA by farmers significantly enhanced the SQI compared to conventional tillage, confirming our second hypothesis. The inclusion of hydraulic conductivity, water holding capacity, dehydrogenase activity, glucosidase activity, available Fe, and available Cu in the minimum data set highlights their importance as key indicators for computing the SQI under the rice–wheat cropping system adopted by farmers in the northwestern IGPs. These indicators can serve as early warnings for changes in soil quality. However, the extent of their utility as influential parameters of soil quality could be limited as they are soil-specific, and their relevance may vary depending on local soil conditions. The significant positive relationship between system productivity (measured as rice equivalent yield and wheat yield) and SQI indicates sustainable crop production under CA. This correlation suggests that SQI can be a valuable tool for predicting crop yields in the rice–wheat system, aiding farmers in better management and decision-making. Based on our findings, we recommend that farmers practice CA for more than 8 years to fully exploit its beneficial effects on soil health and nutrient availability. This extended duration allows the positive impacts of CA to become more pronounced. To maintain soil health and achieve sustainable productivity along with environmental security, it is recommended that farmers adopt long-term CA-based rice–wheat crop rotation.

## Data Availability

The datasets used and/or analyzed during the current study are available from the corresponding author on reasonable request.
